# Impact of early blood purification on serum inflammatory mediators and hemorheology in severe acute pancreatitis

**DOI:** 10.1186/s41065-025-00538-w

**Published:** 2025-09-29

**Authors:** Jing Xiao, Bo Li

**Affiliations:** Department of Critical Care Medicine, The Affiliated Women’s and Children’s Hospital, School of Medicine, Chengdu Women’s and Children’s Central Hospital, University of Electronic Science and Technology of China, Chengdu, 611731 Sichuan Province China

**Keywords:** Severe acute pancreatitis, Blood purification, Inflammatory mediators, Hemorheology, Prognosis

## Abstract

**Background:**

Patients with severe acute pancreatitis (SAP) often experience systemic inflammatory responses and microcirculatory disturbances, for which existing treatments have limited intervention effects.

**Objectives:**

It aimed to investigate the impact of early blood purification on serum inflammatory mediators, hemorheological parameters, and clinical prognosis in patients with SAP.

**Methods:**

120 patients with SAP were randomly grouped: observation group (OG) (routine treatment + early continuous veno-venous hemodiafiltration) and control group (CG) (routine treatment). The time to clinical symptom improvement, hemorheological parameters [whole blood viscosity (WBV), plasma viscosity (PV), hematocrit, and platelet adhesion rate], and inflammatory mediators were compared. Multivariate logistic regression analysis (MLRA) was used to identify prognostic factors.

**Results:**

The OG had markedly shorter times to symptom relief for fever, abdominal pain, and abdominal distension (all *P <* 0.001) and a higher cure rate (*P =* 0.012); The levels of C-reactive protein (CRP), tumor necrosis factor-α (TNF-α), interleukin-6 (IL-6), as well as WBV and PV, were more markedly improved (all *P <* 0.01). Multivariate analysis suggested that peak CRP (OR = 1.01, *P <* 0.001) and peak TNF-α (OR = 1.02, *P =* 0.003) maintained independent predictive value, and all hemorheological parameters were confirmed as independent prognostic factors.

**Conclusion:**

Early blood purification can effectively improve the inflammatory response and hemodynamics in patients with SAP, with its efficacy influenced by multiple factors, including disease severity, intensity of inflammatory response, and hemorheological status.

**Clinical trial number:**

Not applicable.

## Background

Severe acute pancreatitis (SAP) is an acute, life-threatening digestive system emergency with a mortality rate as high as 20%-30%, making it one of the most common acute abdominal conditions in clinical practice [1, 2]. The pathophysiology of this disease is complex, primarily involving autodigestion of the pancreas, excessive activation of the inflammatory response, and the development of multiple organ dysfunction syndrome (MODS). In the early stages of the disease, abnormal activation of pancreatic enzymes within the pancreatic acinar cells leads to autodigestion of the pancreas and surrounding tissues, while also triggering systemic inflammatory response syndrome (SIRS). Large amounts of inflammatory mediators are released into the bloodstream, creating a “cytokine storm” that further exacerbates tissue damage and organ failure [3]. Additionally, patients with SAP often experience severe hemorheological disturbances, such as increased blood viscosity, microcirculatory impairment, and coagulation dysfunction, all of which contribute to disease progression and adverse outcomes. Therefore, effectively regulating the inflammatory response and improving hemorheological status have become key issues in the early treatment of SAP.

Currently, the treatment of SAP mainly includes fluid resuscitation, analgesia, nutritional support, antibiotic administration, and management of complications, but specific treatments targeting excessive inflammatory responses and microcirculatory disturbances are still limited [4, 5, 6]. While fluid resuscitation can correct hypovolemia, excessive fluid administration may exacerbate tissue edema and abdominal compartment syndrome. Analgesic treatment, although effective in relieving severe pain, has limited impact on the underlying pathological processes. Nutritional support helps meet the body’s metabolic needs but does not directly improve microcirculatory disturbances. Antibiotic use, while preventing secondary infections, may introduce new problems such as drug resistance and intestinal dysbiosis [7]. In comparison, blood purification technology has demonstrated unique therapeutic advantages. Blood purification is a technique that removes pathogenic substances from the blood through extracorporeal circulation. Currently, it is widely used in critical conditions such as acute kidney injury, sepsis, and severe pancreatitis. Its core advantages lie in rapidly clearing inflammatory mediators, metabolic waste, and toxins from circulation while regulating fluid balance and internal homeostasis, thereby interrupting the vicious cycle of pathological damage. Regarding treatment costs, this technology incurs higher per-session expenses than conventional therapies (approximately 3–4 times the cost of standard treatment for the high-volume hemofiltration used in this study) due to specialized equipment, consumables, and operational requirements. However, for high-mortality conditions like severe pancreatitis, its long-term benefits—including improved prognosis and reduced hospitalization—may partially offset short-term costs. Clinical application should be comprehensively evaluated based on disease severity and available medical resources [8]. This dual intervention characteristic makes it an important option for breaking through the current treatment bottleneck and provides an ideal research entry point for exploring the impact of early intervention on disease outcomes, which is the main reason for focusing on blood purification technology in this study.

The early treatment of SAP still faces the dual challenges of regulating the inflammatory storm and improving microcirculation, and the application value of blood purification technology has not yet been fully clarified. This study aims to systematically evaluate the dynamic changes in serum inflammatory mediators and hemorheological parameters in patients with SAP following early blood purification and to explore their association with clinical prognosis. The levels of inflammatory factors, the degree of improvement in hemorheology, and short-term prognostic indicators were compared between the early intervention group and the routine treatment group. A multivariate regression model was adopted to identify key factors affecting mortality and organ function recovery. This study hopes to provide a scientific basis for individualized treatment decisions in SAP, optimize the timing and protocols for the clinical application of blood purification technology, and thereby improve patient survival outcomes.

## Materials and methods

### Study subjects

This study employed a combined retrospective screening and prospective enrollment approach for participant selection. The detailed procedure was as follows: First, we retrieved medical records of SAP patients admitted to Chengdu Women’s and Children’s Central Hospital between August 2022 and March 2025, followed by preliminary screening based on clinical diagnostic criteria. Subsequently, strict selection was performed according to predefined inclusion and exclusion criteria, with non-qualifying cases excluded to finalize the study cohort.

The sample size was determined based on the primary outcome measure (poor prognosis, defined as failure to achieve cure or improvement within two weeks of treatment, i.e.,“ineffective” status) and requirements for multivariate logistic regression analysis. Referring to previous studies indicating a 20%-25% incidence of poor prognosis in SAP patients, and considering 10 potential prognostic variables (e.g., Acute Physiology and Chronic Health Evaluation (APACHE) II score, peak inflammatory factor levels), we calculated the sample size according to logistic regression principles (requiring 10–15 events per variable). This yielded a minimum requirement of 100 cases to ensure statistical power (α = 0.05, β = 0.2), with 120 patients ultimately enrolled to enhance result stability. All study protocols were reviewed and approved by the Chengdu Women’s and Children’s Central Hospital ethics committee, and written informed consent was obtained from the patients or their legal representatives.

Inclusion criteria: (1) 18–75 years old; (2) time from onset to admission < 72 h; (3) at least one organ dysfunction within 48 h of onset; (4) complete clinical data; (5) APACHE II score ≥ 8. Exclusion criteria: (1) history of chronic pancreatitis or partial pancreatectomy; (2) other blood purification treatments before admission; (3) active bleeding or complications requiring surgical intervention; (4) terminal illness (expected survival < 3 months); (5) malignancy or immune system diseases.

Using a computer-generated block randomization method, subjects were allocated in a 1:1 ratio to the OG (early blood purification) and the CG (routine treatment), with 60 cases in each group. The random allocation scheme was implemented by an independent statistician using the sealed envelope method.

### Treatment methods

The CG received the standard treatment protocol, which included goal-directed fluid resuscitation management, with initial rapid infusion of lactated Ringer’s solution followed by adjustment of the infusion rate based on hemodynamic parameters; a multimodal analgesia regimen, including continuous infusion of fentanyl via patient-controlled analgesia pump combined with intravenous injection of flurbiprofen ester; early initiation of enteral nutrition support, with gradual increase in the infusion of short-peptide nutritional formulations via nasojejunal tube; prophylactic use of imipenem/cilastatin for anti-infective treatment; and routine monitoring of intra-abdominal pressure changes.CG did not receive routine anticoagulation therapy, with targeted interventions administered only in cases of confirmed coagulation abnormalities.

The OG, in addition to the standard treatment, initiated high-volume hemofiltration within 12 h of admission. The Prismaflex blood purification system (Shanghai Jumu Medical Devices Co., Ltd., China) was used in combination with a large-area AN69ST membrane filter, with vascular access established via a central venous double-lumen catheter. Treatment parameters were set to high blood flow and replacement fluid flow rates, using bicarbonate buffer solution and an unfractionated heparin anticoagulation regimen to maintain the target coagulation time. The treatment lasted for 72 h, during which a professional blood purification team closely monitored treatment parameters, coagulation function, and inflammatory markers, and adjusted the treatment plan in a timely manner. Strict fluid management and complication prevention measures were implemented for all subjects during the treatment period to ensure the safety and effectiveness of the treatment.

### Observation indicators

(1) Collection of baseline data of the subjects: age, gender, body mass index (BMI), APACHE II, sequential organ failure assessment (SOFA), etiology (biliary/alcoholic/other).

(2) Recording of symptom resolution times in the subjects (fever, abdominal pain, abdominal distension, nausea and vomiting). Symptom resolution was defined as complete disappearance of relevant symptoms persisting for > 48 h. For cases with symptom recurrence, the timepoint of final disappearance with sustained 48-hour resolution was recorded. No cases of symptom recurrence after initial resolution were observed in this study.

(3) Clinical efficacy evaluation of the subjects: cure, improvement, and no effect. Cure was defined as complete disappearance of clinical symptoms, normalization of laboratory indicators, and visible absorption of pancreatic inflammation on imaging studies; improvement was characterized by visible improvement in clinical symptoms, ≥ 50% improvement in laboratory indicators compared to baseline, and partial absorption of inflammation on imaging studies; no effect was defined as no improvement or worsening of clinical symptoms, no improvement or worsening of laboratory indicators, and persistent or progressive inflammation on imaging studies. All efficacy assessments were independently completed by two senior physicians using a double-blind method, and any discrepancies were resolved by a third senior physician.

(4) Serum inflammatory mediators (C-reactive protein (CRP), tumor necrosis factor-α (TNF-α), interleukin-6 (IL-6)) of the subjects prior to treatment, 1 week after treatment, and 2 weeks following remedy. Venous blood samples were collected: prior to remedy, 1 week following remedy, and 2 weeks following remedy. ELISA kits (Wuhan Moshake Biotechnology Co., Ltd., China) were used to strictly follow the operating procedures for detection. All tests were completed within 2 h after sample collection, with centrifugation and separation at -80℃ for subsequent testing.

(5) Hemorheological parameters [whole blood viscosity (WBV) values (high shear, low shear), plasma viscosity (PV), hematocrit, and platelet adhesion rate] of the subjects prior to remedy, 1 week following remedy, and 2 weeks following remedy. The German HAAKE RheoStress 6000 rheometer was used by specially trained technicians to measure WBV (high shear 200 s⁻¹ and low shear 5 s⁻¹) and PV at a constant temperature of 37℃. Hematocrit was measured using the microcentrifugation method recommended by the International Committee for Standardization in Hematology, and platelet adhesion rate was detected using the modified Salzman glass bead column method. To ensure data quality, all testing operations were subject to strict internal quality control and regular participation in external quality assessment.

### Statistical methods

Data were processed and analyzed using SPSS 22.0 statistical software. Normally distributed measurement data were expressed as mean ± standard deviation (x̄ ± s), and categorical data were expressed as frequency and percentage (%). For measurement data not normally distributed, the Mann-Whitney test was adopted for contras. For normally distributed measurement data, one-way analysis of variance was adopted. For categorical data, the chi-square test was adopted. Multivariate logistic regression analysis (MLRA) is a statistical method used to examine the relationship between multiple independent variables and a binary dependent variable, enabling evaluation of each variable’s independent effect while controlling for potential confounders.To identify independent risk factors for prognosis, MLRA was performed, including variables such as levels of inflammatory mediators, hemorheological parameters (high shear WBV, platelet adhesion rate), and remedy grouping. *P <* 0.05 was considered statistically significant.

## Results

### Comparison of basic information of subjects

A total of 120 patients with SAP receiving remedy at Chengdu Women’s and Children’s Central Hospital from August 2022 to March 2025 were selected as the study subjects. No statistically significant distinctions were noted in baseline data [including age, gender, BMI, APACHE II score, SOFA score, etiology (biliary/alcoholic/other)] in the subjects (*P >* 0.05), indicating comparability (Table 1).

**Table 1:** Comparison of basic information of participants

Item	OG (*n* = 60)	CG (*n* = 60)	Statistic	*P*
Age (years)	52.36 ± 10.25	53.14 ± 9.87	t = 0.42	0.675
Gender (male/female)	38/22	35/25	χ²=0.38	0.539
BMI (kg/m²)	24.15 ± 3.12	23.87 ± 2.98	t = 0.51	0.613
APACHE II score	12.45 ± 2.36	12.17 ± 2.54	t = 0.61	0.543
SOFA score	4.25 ± 1.32	4.07 ± 1.45	t = 0.71	0.478
Etiology (biliary/alcoholic/other)	32/18/10	30/20/10	χ²=0.21	0.901

### Comparison of symptom resolution time in participants

In Table 2, the resolution time of the main clinical symptoms in the OG was markedly shorter as against the CG (all *P <* 0.001). Specifically, the early blood purification remedy group demonstrated a clear advantage in the speed of symptom relief for fever, abdominal pain, abdominal distension, and nausea and vomiting. Among them, the distinction in abdominal pain symptoms between the groups was the most visible (*t =* 9.34), followed by fever (*t =* 8.12) and abdominal distension (*t =* 7.85). The newly added symptom of nausea and vomiting also suggested a faster relief trend in the OG (*t =* 6.78).

**Table 2:** Comparison of symptom resolution time in participants

Symptoms	OG (*n* = 60)	CG (*n* = 60)	t	*P*
Fever	3.25 ± 1.06	5.42 ± 1.87	8.12	< 0.001
Abdominal pain	4.17 ± 1.32	6.85 ± 2.01	9.34	< 0.001
Abdominal distension	3.89 ± 1.15	5.76 ± 1.64	7.85	< 0.001
Nausea and vomiting	2.98 ± 0.95	4.32 ± 1.28	6.78	< 0.001

### Comparison of clinical efficacy in participants

The results of the Comparison of clinical efficacy (Table 3) suggested that there was a visible distinction in remedy outcomes between the OG and the CG. In terms of cure rate, the OG was markedly higher than the CG (75.00% vs. 53.33%, χ² = 6.27, *P =* 0.012). Although no statistical distinction was noted in the effective rate in the subjects (16.67% vs. 21.67%, χ² = 0.54, *P =* 0.462), the inefficacy rate in the OG was markedly lower as against the CG (8.33% vs. 25.00%, χ² = 6.67, *P =* 0.01).The 3 × 2 contingency table chi-square analysis revealed statistically significant differences in efficacy distribution between groups (χ^2^ = 13.08, *P* = 0.001). Post hoc comparisons with Bonferroni correction demonstrated: the observation group showed higher cure rates (adjusted *P* = 0.014) and lower inefficacy rates (adjusted *P* = 0.012) compared to controls, while response rates showed no significant intergroup difference (adjusted *P* = 0.693).

**Table 3:** Comparison of clinical efficacy in participants

Therapeutic efficacy classification	OG (*n* = 60)	CG (*n* = 60)	χ^2^	*P*
Cure	45(75.00)	32(53.33)	6.27	0.012
Effective	10(16.67)	13(21.67)	0.54	0.462
Ineffective	5(8.33)	15(25.00)	6.67	0.01
Overall analysis of 3 × 2 contingency table			13.08	0.001

### Comparison of inflammatory mediator levels in participants

**Fig. 1:**
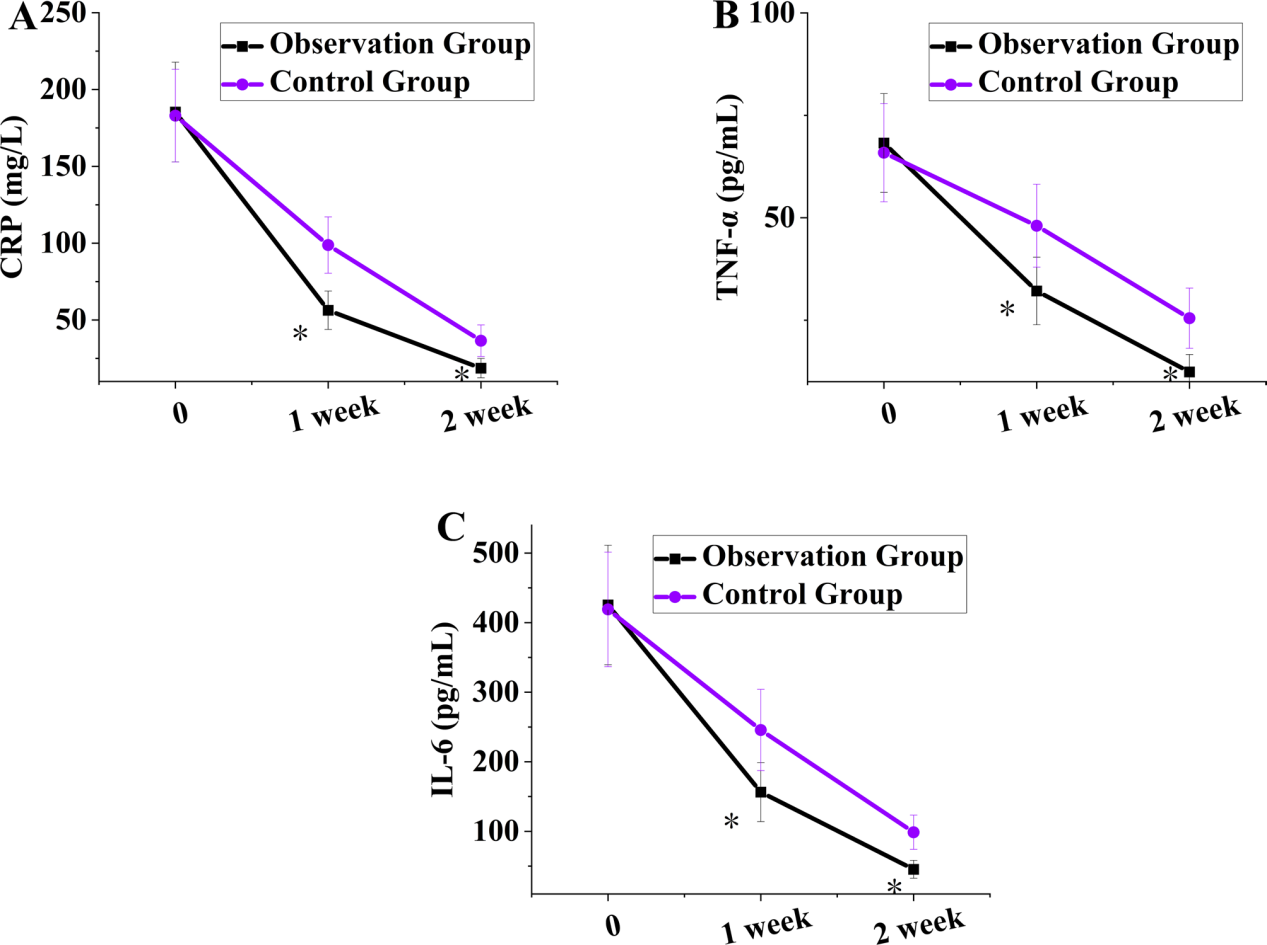
Comparison of inflammatory mediator levels in participants. (**A-C** represent CRP, TNF-α, and IL-6, respectively). Note: * means a visible distinction as against the CG (*P <* 0.05)

The results of dynamic monitoring (Fig. 1) suggested that no visible distinctions were noted in the levels of serum inflammatory mediators in the participants prior to remedy (*P >* 0.05). Following remedy, the reduction in inflammatory markers in the OG was markedly better as against the CG; at 1 week following remedy, the distinctions in all markers among participants were statistically significant (*P <* 0.05). At 2 weeks following remedy, the levels of inflammatory mediators in the OG remained markedly lower than those in the CG (*P <* 0.01).Repeated measures ANOVA was performed to compare serum inflammatory mediator levels between groups at different time points (pre-treatment, 1-week treatment, and 2-week treatment). The results demonstrated statistically significant group, time, and interaction effects (all *P* < 0.01).

### Comparison of changes in hemorheological parameters in participants

**Fig. 2:**
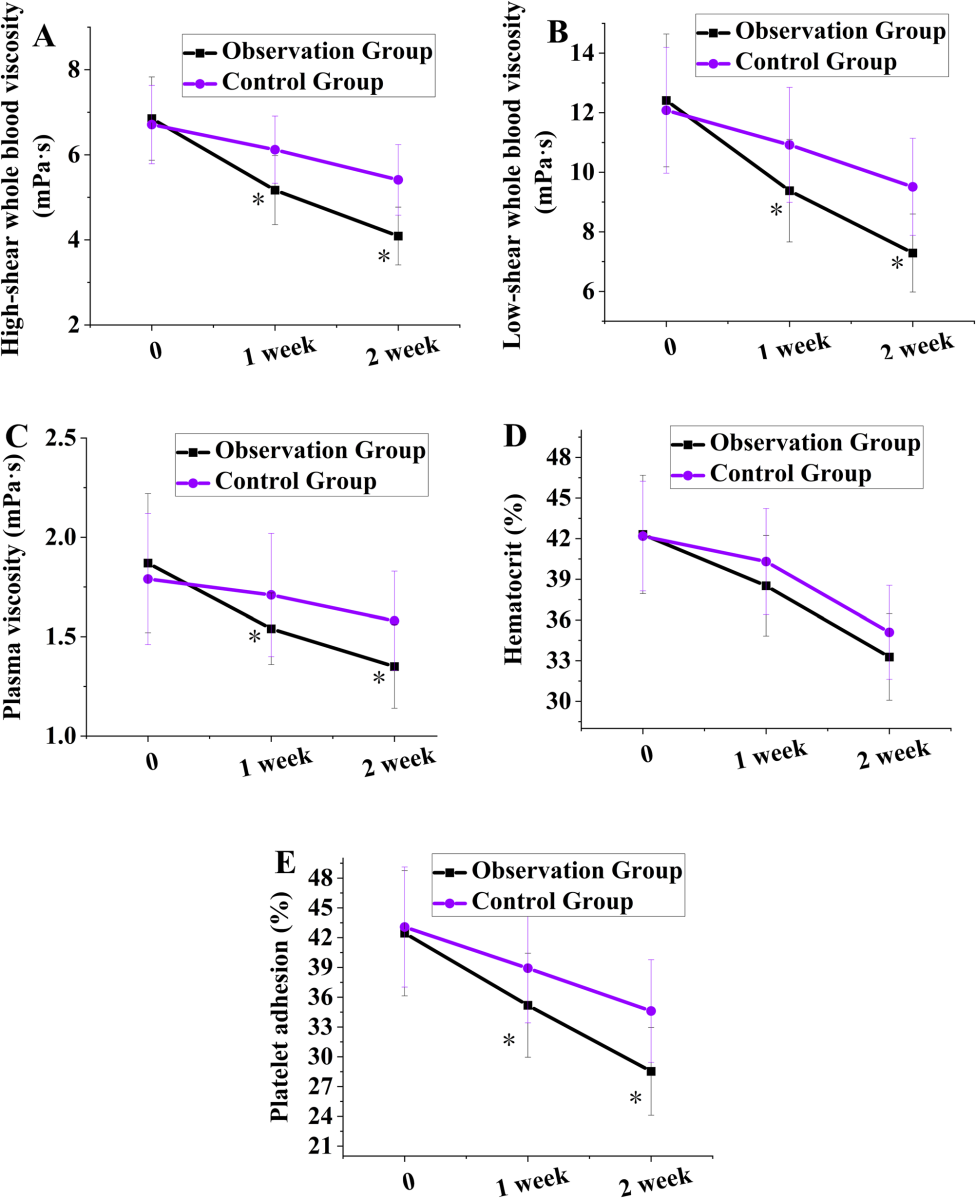
Comparison of changes in hemorheological parameters in participants. (**A-E** represent high-shear WBV, low-shear WBV, PV, hematocrit, and platelet adhesion rate, respectively). Note: * means a visible distinction as against the CG (*P <* 0.05)

The results of hemorheological monitoring (Fig. 2) suggested that the levels of various indicators were comparable among the participants prior to remedy (*P >* 0.05). As the remedy progressed, the OG exhibited a more visible trend of improvement (*P <* 0.01 for intergroup comparison). Repeated measures analysis confirmed that there were visible distinctions between groups and time effects in hemorheological parameters, and an interaction between group and time (all *P <* 0.01). Notably, the improvement in hematocrit did not reach statistical significance between groups (*P >* 0.05), while improvements in other indicators (WBV, PV, and platelet adhesion rate) were statistically significant (*P <* 0.05). Repeated measures ANOVA of hemorheological parameters at different time points revealed significant group, time, and interaction effects (all *P* < 0.01).

### MLRA of prognostic factors

In this study, the binary outcome variable for MLRA was “poor prognosis” (30 cases, prevalence 25.0%). Variables entered into the model included: (1) factors with *P* < 0.1 in univariate analysis (APACHE II score, SOFA score, peak CRP, peak TNF-α, peak IL-6, and hemorheological parameters), and (2) clinically significant variables (treatment group), totaling 10 covariates. The event-to-variable ratio (30:10 = 3:1), combined with Hosmer-Lemeshow test results (*P* = 0.582), indicated no significant overparameterization of the model.

Univariate logistic regression analysis suggested (Table 4) that the APACHE II score (*P <* 0.001), SOFA score (*P =* 0.001), peak CRP (*P <* 0.001), peak TNF-α (*P =* 0.004), as well as hemorheological parameters such as high-shear WBV (*P =* 0.011), low-shear WBV (0.015), PV (0.021), and platelet adhesion rate (0.014) were markedly associated with prognosis. Demographic characteristics (age, gender, BMI) did not show statistical significance (all *P >* 0.05).

After adjusting for confounding factors, MLRA suggested (Table 5) that the APACHE II score (OR *=* 1.51, *P <* 0.001), SOFA score (OR *=* 1.43, *P =* 0.001), peak CRP (OR *=* 1.01, *P <* 0.001), and peak TNF-α (OR *=* 1.02, *P =* 0.003) maintained independent predictive value. All hemorheological parameters (high-shear WBV *P =* 0.009, low-shear WBV 0.013, PV 0.015, platelet adhesion rate 0.012) were confirmed as independent prognostic factors.

**Table 4:** Univariate logistic regression analysis of prognostic factors

Variables	β	SE	Wald χ²	*P*	OR (95%CI)
APACHE II score	0.38	0.09	17.25	< 0.001	1.46(1.23–1.74)
SOFA score	0.34	0.1	11.56	0.001	1.40(1.15–1.71)
Peak CRP (mg/L)	0.01	0	15.32	< 0.001	1.01(1.00-1.02)
Peak TNF-α (mg/L)	0.02	0.01	8.45	0.004	1.02(1.01–1.03)
Peak IL-6 (mg/L)	0	0	3.98	0.046	1.39(1.03–1.88)
High-shear WBV	0.31	0.12	6.45	0.011	1.36(1.07–1.73)
Low-shear WBV	0.27	0.11	5.87	0.015	1.31(1.05–1.63)
PV	0.23	0.1	5.32	0.021	1.26(1.03–1.54)
Platelet adhesion rate (%)	0.04	0.02	5.98	0.014	1.04(1.01–1.08)
Age (years)	0.02	0.01	1.85	0.174	1.02(0.99–1.05)
Gender (male)	0.17	0.19	0.78	0.378	1.19(0.81–1.74)
BMI (kg/m²)	0.03	0.03	1.25	0.264	1.03(0.98–1.09)

**Table 5:** MLRA of prognostic factors

Variables	β	SE	Wald χ²	*P*	OR (95%CI)
Remedy grouping (OG)	-1.3	0.35	13.78	< 0.001	0.27(0.14–0.54)
APACHE II score	0.41	0.1	17.89	< 0.001	1.51(1.24–1.83)
SOFA score	0.36	0.11	10.45	0.001	1.43(1.15–1.78)
Peak CRP (mg/L)	0.01	0	16.32	< 0.001	1.01(1.00-1.02)
Peak TNF-α (pg/mL)	0.02	0.01	9.12	0.003	1.02(1.01–1.03)
Peak IL-6 (pg/mL)	0	0	3.98	0.046	1.00(1.00-1.01)
High-shear WBV	0.33	0.13	6.78	0.009	1.39(1.08–1.78)
Low-shear WBV	0.28	0.11	6.12	0.013	1.32(1.06–1.65)
PV	0.25	0.1	5.89	0.015	1.28(1.05–1.57)
Platelet adhesion rate	0.05	0.02	6.32	0.012	1.05(1.01–1.09)

Note: The model variable adjustment process followed these steps: First, variables showing significance in univariate analysis were included, then stepwise regression (entry α = 0.05, removal α = 0.10) was performed to ultimately retain variables with independent predictive value. This adjustment process satisfied both clinical and statistical criteria for confounding variable control

## Discussion

SAP, as a common critical illness in clinical practice, has always posed a challenge to clinical remedy due to its high mortality rate [9, 10]. In recent years, although supportive treatments have continuously improved, specific interventions targeting excessive inflammatory responses and microcirculatory disturbances remain insufficient. This study, through a prospective randomized controlled design, systematically evaluated the impact of early blood purification on inflammatory mediators and hemorheology in patients with SAP and analyzed key factors affecting prognosis, providing new evidence-based support for clinical practice. The results of this study suggested that early blood purification therapy markedly improved the clinical symptoms of patients with SAP. The OG had markedly shorter times to symptom resolution for fever, abdominal pain, and abdominal distension as against the CG, findings that are consistent with the observed effects of blood purification on the clearance of inflammatory factors in clinical practice. Traditional treatments primarily alleviate symptoms through fluid resuscitation and organ function support, while blood purification intervenes at the source of the inflammatory cascade, which may be the key mechanism for the rapid improvement of symptoms [11]. It is particularly noteworthy that abdominal pain, the most prominent symptom of SAP, suggested the most visible distinction in resolution time, suggesting that blood purification may more effectively relieve pain by reducing local pancreatic inflammation, edema, and ischemia. In terms of clinical efficacy, the OG had a markedly higher cure rate and a markedly lower inefficacy rate, results that surpass the expected outcomes of conventional remedy. Of note, the CG showed a slightly higher response rate than the observation group (21.67% vs. 16.67%), though this difference was not statistically significant - potentially attributable to limited sample size and interindividual heterogeneity in conventional treatment response. Clinically, “response” primarily reflects partial improvement in symptoms and parameters, whereas this study focused on “cure” representing fundamental disease resolution. The observation group’s significantly higher cure rate and lower treatment failure rate more robustly demonstrate early blood purification’s therapeutic impact on SAP progression, rendering the marginal difference in response rates inconsequential to overall treatment superiority.In clinical practice, the remedy outcomes of SAP are often closely related to the duration of the disease and the degree of organ damage. However, this study shows that early intervention with blood purification can change the natural course of the disease. It is particularly noteworthy that although there was no statistical distinction in the effective rate of participants, the visible increase in the cure rate and the visible decrease in the inefficacy rate in the OG suggest that blood purification may change the key pathological aspects of the disease, leading to fundamental improvements in more patients rather than partial relief [12].

The dynamic monitoring results of inflammatory mediators revealed the core mechanism of blood purification. CRP, TNF-α, and IL-6, as important indicators reflecting systemic inflammatory responses, suggested a clear temporal correlation between their rapid decline and the improvement of clinical symptoms. Under traditional remedy, the levels of inflammatory mediators often show a slow downward trend, while the rapid decline in the blood purification group suggests that this remedy may interrupt the vicious cycle of the cytokine storm [13, 14]. It should be noted that while blood purification may non-specifically eliminate some potentially beneficial mediators during the clearance of excessive pro-inflammatory factors, our findings and clinical experience demonstrate that in SAP patients, the “cytokine storm”-induced hyperinflammatory response represents the core pathological mechanism driving tissue damage and organ dysfunction. In this context, the detrimental effects of accumulated pro-inflammatory mediators far outweigh the potential impact of limited beneficial mediator clearance.Furthermore, the high-volume hemofiltration mode and AN69ST membrane filters employed in this study exhibit relative selectivity, preferentially removing larger molecular weight inflammatory factors (e.g., TNF-α, IL-6). Through dynamic monitoring of inflammatory markers and organ function during treatment, purification parameters could be promptly adjusted to minimize excessive clearance of beneficial mediators. This approach ultimately achieved the primary therapeutic objectives of mitigating inflammatory injury and improving clinical outcomes.It is particularly noteworthy that TNF-α, as an upstream mediator in the inflammatory cascade, may play a key role in suppressing subsequent inflammatory responses through its early decline, providing important clues for understanding the mechanism of action of blood purification. The improvement of hemorheological parameters provided another perspective for understanding the therapeutic effect. The visible reduction in high-shear and low-shear WBV reflects the direct effect of blood purification on improving microcirculatory disturbances. Although traditional clinical treatments also focus on fluid resuscitation and blood dilution, the improvement in hemorheology is often limited. The comprehensive improvement of hemorheological parameters in the OG in this study, especially the decrease in platelet adhesion rate, suggests that blood purification may improve microcirculation through multiple mechanisms, including the clearance of inflammatory mediators that promote red blood cell aggregation and platelet activation. It is worth noting that the improvement in hematocrit was not visible, which may be related to the fact that all participants received standardized fluid resuscitation, also suggesting that the effect of blood purification on hemorheology is mainly achieved through other mechanisms [15]. The analysis of prognostic factors provided a basis for individualized remedy. Multivariate regression analysis confirmed that remedy group was the strongest independent prognostic factor, with its protective effect exceeding traditional prognostic indicators such as the APACHE II score. This finding challenges the traditional concept of relying solely on disease severity to predict prognosis and emphasizes the importance of early intervention. All hemorheological parameters maintained independent predictive value, indicating the key position of microcirculatory disturbances in the prognosis of SAP. This result is in line with the clinical observation that even with good control of inflammation, some patients still experience organ dysfunction due to microcirculatory disturbances [16].

Early blood purification suggested advantages in multiple aspects. Traditional treatments mainly rely on passive support waiting for the body to repair itself, while blood purification adopts a strategy of actively removing pathogenic factors. This shift in remedy philosophy may be the fundamental reason that the OG achieved better therapeutic effects [17]. The dual regulatory effect of blood purification on inflammatory mediators and hemorheology enables it to target the two core pathological aspects of SAP simultaneously, which may be the key to surpassing single anti-inflammatory or circulatory improvement remedy. The results of this study have important implications for clinical practice. First, the timing of early intervention is crucial; intervention before the occurrence of organ dysfunction may achieve the best results. Second, individualized adjustment of remedy parameters deserves attention, such as dynamically adjusting the intensity and duration of purification based on inflammatory mediator levels and hemorheological parameters [18]. Finally, a comprehensive evaluation system combining multiple assessment indicators (including clinical symptoms, laboratory tests, and imaging) is more accurate in reflecting therapeutic effects than a single indicator. In terms of safety, this study did not find any serious adverse reactions related to blood purification, consistent with the application experience of this technology in other critical illnesses. Considering that patients with SAP often have coagulation dysfunction and hemodynamic instability, close monitoring and timely adjustment of parameters during remedy may be the key to ensuring safety [19]. This finding supports the inclusion of early blood purification as a routine remedy option for SAP in medical centers with the necessary conditions.

This study has several methodological limitations that warrant clarification: (1) The relatively limited sample size (*n* = 120) may reduce the ability to detect rare adverse effects and statistical power, particularly affecting the reliability of subgroup analyses (e.g., efficacy differences among SAP etiologies); (2) Despite randomized allocation, blinding of patients and treating clinicians was impossible due to the invasive nature of blood purification in the observation group, potentially introducing assessment bias; (3) Although key covariates like APACHE II scores were adjusted for, unidentified confounders (e.g., baseline disease severity, concomitant medication variations) may influence results interpretation. Additionally, certain treatment outcomes showed no significant improvement - notably, hematocrit changes did not differ statistically between groups, suggesting limited efficacy of early blood purification for hemoconcentration, potentially related to standardized fluid resuscitation in both groups. This also indicates that microcirculatory improvement involves multiple mechanisms beyond purification alone. These limitations highlight the need for validation through larger, multicenter blinded trials. Furthermore, the cohort predominantly comprised biliary and alcoholic etiologies, with limited hyperlipidemic cases, potentially restricting generalizability to lipid-induced pancreatitis. Gulumsek et al. [20] demonstrated no superiority of plasma exchange over conventional therapy in hypertriglyceridemia-induced pancreatitis, suggesting potential etiology-dependent treatment responses. Thus, our findings require further validation in hyperlipidemic SAP, emphasizing the need for future studies with adequate subgroup representation to elucidate etiology-specific efficacy differences [21, 22].

## Conclusion

This study confirmed through a prospective randomized controlled trial that early blood purification therapy markedly improves the clinical prognosis of patients with SAP. The results suggested that combining early high-volume hemofiltration with conventional supportive remedy can more rapidly relieve clinical symptoms in patients, markedly increase the cure rate, effectively reduce systemic inflammatory responses, and improve microcirculatory disturbances. Multivariate analysis indicated that early blood purification is an independent protective factor for prognosis, with effects superior to traditional prognostic assessment indicators. The study provides important insights for clinical practice: for patients with SAP who meet the inclusion criteria, initiating blood purification therapy within 12 h of onset may achieve the best therapeutic effects. During remedy, close monitoring of inflammatory indicators and hemorheological parameters is essential, which not only helps assess therapeutic effects but also provides a basis for adjusting remedy plans. Considering the good safety profile of this remedy, it is recommended to include it as an important component of comprehensive remedy for SAP in medical centers with the necessary conditions.

## Data Availability

All data generated or analyzed during this study are included in this published article.
